# Increased hematogones in an infant with bicytopenia and leucocytosis:a case report

**DOI:** 10.1186/1757-1626-3-75

**Published:** 2010-03-04

**Authors:** Kiran Agarwal, Meenu Aggarwal, Vikas Kumar Aggarwal, Meenu Pujani, Manupriya Nain

**Affiliations:** 1 Department of Pathology, Lady Hardinge Medical College, New Delhi -1, India; 2 Department of Pediatrics, Kalawati Saran Children Hospital, New Delhi-1, India

## Abstract

Hematogones are the normal bone marrow constituents of bone marrow in children and their number decreases with age. As hematogones can resemble malignant lymphoblasts by their morphologic features and by expression of an immature B-cell phenotype, an accurate distinction of hematogone-rich lymphoid regeneration from leukemic lymphoblasts is critical for patient care. The increased number of hematogones had been reported in the bone marrow of children recovering from chemotherapy, aplastic conditions, other forms of bone marrow injury, infections like Cytomegalovirus, HIV and immune thrombocytopenia disorders. We describe here a case of one and half month old male infant with bicytopenia and leucocytosis associated with increased hematogones in the bone marrow due to an unknown probable viral infection.

## Introduction

Hematogones (B-lymphocyte progenitor cells) along with mature B lymphocytes are the normal bone marrow constituents of pediatric bone marrow and their number decreases with age[1]. Despite the frequency of hematogones, it is difficult to determine normal reference ranges for them, yet 5% or more is generally considered increased[2]. They show a spectrum of maturation bridging those of mature lymphocytes and neoplastic lymphoblasts[3]. Hematogones can resemble malignant lymphoblasts by their morphologic features and by expression of an immature B-cell phenotype, an accurate distinction of hematogone-rich lymphoid regeneration from leukemic lymphoblasts is hence critical for patient care. The increased number of hematogones had been reported in the bone marrow of children recovering from chemotherapy, aplastic conditions, other forms of bone marrow injury, infections like Cytomegalovirus, HIV and immune thrombocytopenic disorders [2-5]. However, increase in number of hematogones due to unknown viral infections had been rarely reported in the literature[1]. We describe here a case of one and half month old male infant with bicytopenia, leucocytosis associated with increased hematogones in the bone marrow due to an unknown infection.

## Case Report

**Figure 1:**
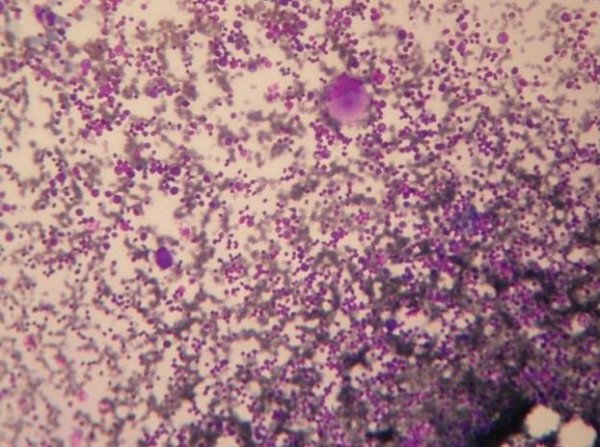
**Cellular Marrow with Normal Megakaryocytes (400×)**.

**Figure 2:**
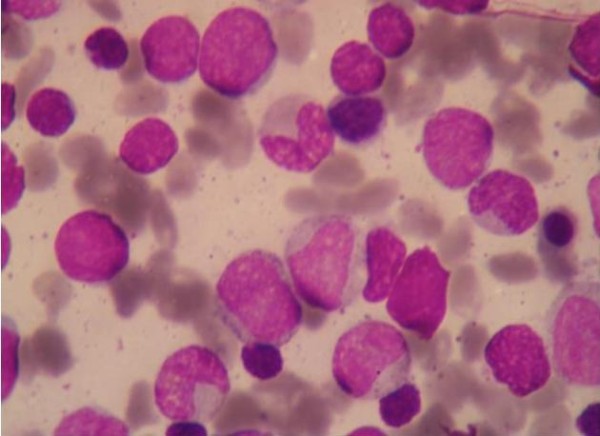
**Small and Large Hematogones along with nucleated RBCs and myeloid precursors (1000×)**.

A one and half month old term infant was admitted to the Kalawati Saran Children Hospital with fever and petechiae all over the body for one day. There was no history of any major bleed, organomegaly or lymphadenopathy. His weight and length were 4.2 Kg and 37 cm respectively (normal for age). His head circumference was 37 cm (normal for age). Child was pale, but was feeding well. His eye and fundus examination were normal. A complete blood count revealed a Total Leukocyte count of 22300/cumm, Platelet count of 4000/cumm and Hemoglobin of 5.8 g/dl. Mean corpuscular volume was 89.5fl and Mean corpuscular hemoglobin was 30.4pg. Since, patient had severe anemia, leukocytosis and thrombocytopenia, a provisional clinical diagnosis of Acute Leukemia was made and a peripheral smear along with bone marrow aspirate was sent. Peripheral blood smear showed leukocytosis, Normocytic Normochromic red blood cells and mild anisocytosis. Platelets were reduced. Differential Leukocyte count was Neutrophil-28%, Lymphocyte-60%, Eosinophils 02%, Monocytes-08%, Stab-02%. Bone Marrow Aspirate smears were cellular with presence of lymphocytosis varying from mature lymphocytes to immature lymphocytes (figure 1). These Immature Lymphocytes were 1-3 times the size of a small mature lymphocyte with scant agranular pale blue cytoplasm, high Nucleo-cytoplasmic ratio, condensed to finely opened up chromatin. Occasional cell showed 0-1 indistinct nucleoli (figure 2). Erythroid series showed normoblastic reaction. Myeloid series showed normal maturation. There was no increase in number of Megakaryocytes and they were also normal in morphology. Myelogram was Immature Lymphocytes-40%, Myelocytes-09%, Metamyelocytes-07%, Stab-13%, Neutrophils-08%, Eosinophils-02%, Lymphocytes-3%, nucleated RBC-18%. Impression of Leucocytosis with thrombocytopenia with normocytic normochromic anemia with presence of immature lymphocytes was given and Immunophenotyping by Flow Cytometry was advised to rule out Leukemia.

For immunophenotyping 3-colour Flow Cytometry was used. Gating using forward side scatter and CD-45 side scatter analysis. A limited antibody panels consisting of CD45, CD10, CD19, CD20, CD34, CD38, CD56 and HLA-DR were used to confirm the nature of these immature lymphocytes. The bone marrow specimen showed a small cell cluster expressing moderately dim CD45, the intensity of which was slightly lesser than the normal lymphocyes on the SSC/CD45 dot plot with low SSC. These cells comprised 20-22% of the cells in the sample and displayed heterogenous FSC indicating variability in size, They expressed dim CD19 with variable CD10 (69%). The expresssion of CD20 was markedly heterogenous varying in intensity from negative to bright producing a "J- shaped trail pattern" on CD10/CD20 dot plot. These cells expressed moderately bright CD38 (86%) and dim HLA-DR (84%). A tiny fraction of these cells (1%) showed expression of CD 34. They did not express CD56.

Based on morphology and immunophenotyping features those immature lymphocytes were diagnosed to be Hematogones. Serum sent for TORCH and Human Immunodeficiency Virus (HIV) was negative for antibodies.

Hence, a diagnosis of thrombocytopenia with leucocytosis with increased hematogones in the bone marrow due to unknown probable viral infection was given.

Patient was kept in close follow up and was given hematinics. No antibiotics, steroids or immunoglobins were given. His platelet count and Hemoglobin recovered to 2 lacs and 10.4 g/dl respectively. His TLC came down to 9,000/cumm. Patient is still doing fine 6 months after the initial diagnosis and has not developed any other signs and symptoms.

## Discussion

Hematogones are the normal constituents of bone marrow in newborns and infants upto several weeks[1]. Due to their morphological similarity to blasts, they have also been a diagnostic dilemma and their presence can lead to erroneous diagnosis. On morphology, Hematogones vary in shape from 10 to 20 μ in diameter, with smaller cells predominating. The nucleus is round or oval and exhibits one or more indentations or shallow clefts. The nuclear chromatin is condensed but homogeneous. Nucleoli are absent or small and indistinct. Cytoplasm is generally scant but when present, is moderately to deeply basophilic and devoid of inclusions, granules, or vacuoles. There is often a spectrum of size and cytologic features that blended with those of mature lymphocytes[6]. In our case also, Bone Marrow Aspirate smears showed 40% immature lymphocytes with morphology as described above. Hematogones can resemble malignant lymphoblasts by their morphologic features and by expression of an immature B-cell phenotype[4,7]. Thus, morphological and immunophenotypic features of hematogones require a careful differential diagnosis to rule out ALL especially when concomitant anemia and/or thrombocytopenia and/or splenomegaly are observed. Single and 2-color flow cytometry do not reliably differentiate hematogones from leukemic lymphoblast. However, appropriately applied, 3- and 4-color multiparametric flow cytometry are reported to distinguish between these cell populations in nearly all instances[6]. In multiparameter flow cytometric studies, they express a complete spectrum of antigen expression that defines the normal evolution of B-lymphocyte lineage. The earliest recognizable B-lineage precursors express TdT and the progenitor cell marker CD34 in combination with CD38, CD19, high levels of CD10 and low levels of CD22; they lack CD20 (stage I). There is a stage where CD10/CD19 are co-expressed while kappa and lambda are completely negative; probably this is stage II hematogones. In next stage there is down-regulation of TdT and CD34 completely and CD10 partially, prior to progressive up-regulation of CD20. CD22 is also increased slightly as CD20 is up regulated (stage 3). Lastly CD10 is down-regulated completely. The cells in which CD10 is completely down regulated are considered mature B-lymphocytes. Depending on their stage of maturation of, markers could be: CD10, CD19, CD34 positive cells, CD10, CD19 positive cells or CD19, CD22 positive ones[8]. That is why there is a different range of morphology from very immature (blast like) to more mature forms as was also seen in the present case. In contrast, the ALL samples consistently express a more immature, but homogeneous, immunophenotype, with the majority of cases expressing TdT, CD34, or both[7].

Caldwell and associates in a study of 45 healthy children and adults found that the number of marrow B-cell precursors is greater in children than adults and declines with age[2]. Lucio and coinvestigators studied 39 normal bone marrow samples and found a shift in B-cell populations from predominantly immature precursors in individuals aged less than 15 years to mostly more mature B-cell immunophenotypes in patients 15 years and older. It was reported that in infants less than 2 years of age, hematogones averaged 9%, by 2 - 5 years the percent dropped to 3.9% and in patients more than 50 years of age, the average was less than 1% [7]. Increased hematogones had also been observed in a variety of clinical conditions like ITP, regenerating marrow of treated ALL, after autologous bone marrow transplantation in acute myeloid leukemia and in viral infections commonly due to CMV or HIV[1,3,6]. These data suggest that a mechanism involving immuno-stimulation of the lymphoid population probably underlies the increased proportion of lymphocytes expressing the immature B phenotype. Alternatively, this phenomenon could be the result of the regeneration of the stem cell compartment after transient damage involving the platelet compartment[9]. In general the presence of hematogones in the bone marrow of ITP and/or other benign conditions, may potentially cause diagnostic problems because of the morphologic and immunophenotypic features they commonly share with neoplastic B cell precursor lymphoblasts.

Also injury due to drugs and an unknown viral infection other than CMV, HIV as a cause promoting an immunological response leading to the regeneration of bone marrow and expression of the immature B phenotype cannot be excluded. Very few cases had been described in the literature in which children had clinical picture of cytopenias with increased hematogones in the bone marrow but no probable cause could be isolated[1]. It was postulated that an unknown viral infection might be responsible for these. In the present case, TORCH and HIV were negative and no other cause for bicytopenias with leukocytosis could be isolated. Flow cytometry revealed presence of hematogones. Hence, a diagnosis of increased hematogones due to an unknown probable viral infection was made. Patient is also doing well six months after the initial diagnosis. So, one should be cautious in reporting leukemia with such a clinical and haematological picture and it must be kept in mind that viral infections other than CMV and HIV can also lead to increase in hematogones.

To conclude one should not hurry in making a diagnosis of leukemia in neonates or infants even if the clinico-hematological profile favours it and no definite cause for increased immature B lymphocytes is isolated. Flow cytometry to rule out hematogones is a must in these cases.

## Consent

Written informed consent was obtained from the patient for publication of this case report and accompanying images. A copy of the written consent is available for review by the journal's Editor-in-Chief.

## Competing interests

None of the authors have any financial or non-financial potential conflicts of interest. There is no funding from nor any shares from any organisation that stand to gain or lose from the publication of this case, holding patents related to the case, nor any other competing interests.

## Authors' contributions

KA - In making final diagnosis, writing the manuscript and correcting the final draft. MA - Helped in reaching diagnosis and writing the manuscript. VKA - Provided clinical details and follow up of patient. MP - Helped in taking out references and making diagnosis. MN - Helped in taking out references and follow up of patient. All authors have read and approved the final manuscript.
